# Evaluating medical service performance of hospitals in Sichuan Province, China: exploratory factor analysis and hierarchical clustering analysis based on diagnosis-related groups

**DOI:** 10.3389/fpubh.2025.1617945

**Published:** 2025-07-24

**Authors:** Xuedong Liu, Jian Cao, Ruyu Ge, Ou Jiang

**Affiliations:** ^1^Department of Medical Administration, The First People’s Hospital of Neijiang, Neijiang, China; ^2^School of Public Health, Chongqing Medical University, Chongqing, China

**Keywords:** DRG_S_, exploratory factor analysis, hierarchical clustering analysis, Donabedian, hospital, performance evaluation

## Abstract

**Objective:**

This study aims to evaluate hospital medical service performance in Sichuan Province, China.

**Methods:**

A total of 306 secondary and tertiary general hospitals were included in the analysis. A comprehensive evaluation model was developed using exploratory factor analysis (EFA) based on diagnosis-related groups (DGR_S_) indicators to assess medical service performance. Indicators were determined within the Donabedian structure-process-outcome (SPO) framework. Hierarchical clustering analysis (HCA) was applied to categorize hospitals into performance clusters, and the Kruskal-Wallis H test was used to compare disparities in performance characteristics across clusters.

**Results:**

The comprehensive evaluation revealed that all top 10 hospitals were tertiary general hospitals (TGHs), with 40.00% located in the Chengdu region. Conversely, the bottom 10 hospitals were exclusively secondary general hospitals (SGHs), predominantly concentrated in northeastern Sichuan. TGHs were classified into three clusters: “Excellent” (30.83%), “Middle” (57.14%), and “Inferior” (12.03%), while SGHs were categorized as “Excellent” (26.01%), “Middle” (69.94%), and “Inferior” (4.05%). For TGHs, the “Excellent” cluster displayed significantly higher performance in case-mix index (CMI), number of DRG_S_ (ND), total weight (TW), and time efficiency index (TEI) compared to the “Middle” and “Inferior” clusters, but performed worst in cost efficiency index (CEI) and mortality of middle and low-risk group cases (MMLRG). For SGHs, “Excellent” cluster hospitals significantly outperformed others in ND and TW, while the “Inferior” cluster performed best in CMI but alarmingly worst in MMLRG.

**Conclusion:**

Significant regional and hierarchical disparities in medical service performance were observed across Sichuan Province, with Chengdu region demonstrating optimal performance. For TGHs, hospitals in the “Inferior” cluster are recommended to enhance their medical ability and efficiency compared to those in the “Excellent” cluster. Conversely, hospitals in the “Excellent” cluster should focus on controlling medical costs compared to those in the “Inferior” cluster. For SGHs, hospitals in the “Inferior” cluster should concentrate on improving medical security and ensuring patient safety compared to those in the “Middle” and “Excellent” clusters.

## 1 Introduction

The evaluation of medical service performance has emerged as a critical research focus, garnering substantial attention worldwide. Over the past few decades, extensive literature has explored various instruments for measuring hospital service quality (1). In 1966, American scholar Avedis Donabedian proposed the classic three-dimensional quality framework, comprising the dimensions of structure, process, and outcome (SPO) to assess healthcare quality (2, 3). In this framework, “structure” denotes the physical settings, provider qualifications, and administrative systems; “process” refers to the delivery components of care; and “outcome” encompasses recovery, functional restoration, and survival (3). Through continuous international scholarly exploration, the Donabedian model has deepened its theoretical connotations and become a globally recognized framework for healthcare quality assessment (2).

In China, scholars have applied the Donabedian framework to construct evaluation systems. For example, Wang et al. developed an index system for evaluating the core competencies of hospital specialist service operation assistants using SPO-based indicators (4). Cai et al. established a single-disease quality management system guided by the SPO model (5). Wang et al. clarified the model’s connotations in Chinese healthcare, defining “structure” as the static configuration and efficiency of institutional resources, “process” as the dynamic quality and efficiency of service operations, and “outcome” as the integrated measurement of structural and process quality (6). This framework has categorized medical ability and security indicators under “outcome” and efficiency indicators under “process” dimensions, forming a theoretical foundation for similar quality assessment studies.

China has implemented SPO-based hospital performance evaluation for years. A significant milestone was the 2019 State Council document entitled “Strengthening Performance Evaluation of Tertiary Public Hospitals” (7). This initiative focused on SPO dimensions relating to hospital management, such as sustainable development, operational efficiency, medical quality, and patient satisfaction (7). Indicators like inpatient workload per physician, physician-to-nurse ratio, and outpatient satisfaction were used to construct a comprehensive evaluation matrix (7). A notable feature of this approach is the use of isolated indicators with assigned weights to form the assessment system.

However, previous studies have identified limitations in using isolated indicators such as average cost, length of stay (LOS), mortality, work efficiency, and workload for performance evaluation (8–10). These indicators are inadequate and inappropriate due to healthcare’s complexity (9), diverse needs, and information asymmetry (11, 12). Such isolated indicators fail to capture the full spectrum of service quality (1, 13), raising concerns about comparability and comprehensiveness (10). One widely recognized approach is integrating risk adjustment into evaluation processes (8, 14). Diagnosis-related groups (DRG_S_), a patient classification system developed at Yale University in the 1970s, standardizes healthcare payment and performance assessment by grouping patients with similar clinical causes and treatments (10, 15, 16). A significant application is for performance evaluation. For instance, Vitikainen et al. used two different output grouping systems (Classic and FullDRG) to estimate hospital efficiency (17). Luo et al. utilized DRG_S_ indicators, including case-mix index (CMI), number of DRGs (ND), total weight (TW), cost efficiency index (CEI), time efficiency index (TEI), and mortality of middle and low-risk group cases (MMLRG) to objectively evaluate inpatient performance among tertiary hospitals in Sichuan’s Panxi region (18).

Since its inception, numerous DRG_S_-based evaluation models have been developed. Jian et al. used CEI, TEI, CMI, and inpatient mortality of low-risk group cases (IMLRG) to evaluate inpatient service performance in Beijing (19). Liu et al. adopted CMI, ND, TW, CEI, TEI, and IMLRG to evaluate medical service performance for breast cancer patients in Henan Province (20). Lu et al. evaluated an organ transplant department using similar metrics (21). These models primarily compare inter-hospital performance via DRG_S_ indicators themselves. Contrasts to previous studies, Liu et al. developed two models by combining principal component analysis (PCA), entropy, TOPSIS, and rank sum ratio (RSR) methods based on CMI, ND, TW, CEI, TEI, MMLRG, and hospital case fatality rate (SCFR) to evaluate TGHs performance (10).

Despite great advancements in hospital performance evaluation, literature review has revealed that no studies have integrated exploratory factor analysis (EFA) and hierarchical clustering analysis (HCA) methods based on DRG_S_ indicators within the Donabedian theoretical framework to evaluate hospital medical service performance. Given this gap, our study introduces a novel model combining the two methods based on DRG_S_ indicators within the Donabedian theoretical framework to assess 306 hospitals in Sichuan, China. The findings of this study may inform healthcare management and future research.

## 2 Methods

### 2.1 Hospital determination

**Figure 1:**
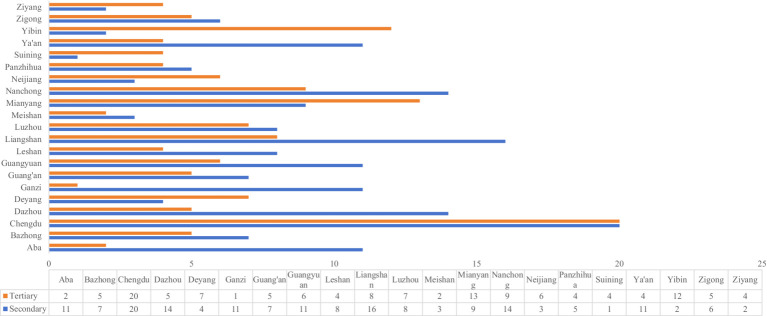
Geographic and hospital-level distributions of the 306 study hospitals.

This study focused on secondary general hospitals (SGHs) and tertiary general hospitals (TGHs) in Sichuan, China. Hospitals specializing in traditional Chinese medicine, traditional Chinese medicine and Western medicine hospitals, and specialized hospitals were excluded. To holistically assess the disparities in medical service performance across hospital levels, all SGHs and TGHs registered in the Sichuan Health Data Analysis and Decision Support Cloud Platform (SHDADSCP) (22) were selected as the study hospitals. This resulted in a total sample size of 306 hospitals, including 173 SGHs and 133 TGHs. These hospitals are distributed across all 21 municipalities in Sichuan. Detailed geographic and hospital-level distributions of the study hospitals are displayed in Figure 1.

### 2.2 Indicator selection

In 2014, the former Health and Family Planning Commission of Sichuan Province introduced the “Front Page of Medical Records (FPMR) (2014 edition)” (23), a standardized format for collecting inpatient medical data across the region. This data included demographic characteristics, diagnosis and treatment information, and medical expenses (24). In 2024, the SHDADSCP analyzed the FPMR data from 306 hospitals and developed a comprehensive evaluation matrix. This matrix consisted of three primary indicators and seven secondary indicators (22). In reference to relevant studies (23, 25, 26), this study selected six indicators for evaluation: CMI, ND, TW, CEI, TEI, and MMLRG. Among these, CMI, ND, and TW were considered positive indicators, while CEI, TEI, and MMLRG were negative indicators (27–29). Detailed explanations of these evaluation indicators are illustrated in Table 1.

**Table 1:** Explanations of medical service performance evaluation indicators.

Indicators	Dimensions of indicators	Explanations of indicators	Attribute of indicators
CMI (x_1_)	Medical ability indicators	Higher CMI values in hospitals indicate their advanced medical techniques in treating critically ill and complex patients.	Positive indicators
ND (x_2_)		Higher ND values reflect a hospital’s capacity to provide a broader range of medical service.	
TW (x_3_)		Higher TW values suggest greater output of inpatient service.	
CEI (x_4_)	Medical efficiency indicators	Lower CEI values imply lower costs for treating similar diseases.	Negative indicators
TEI (x_5_)		Lower TEI values indicate shorter hospital stays for treating similar diseases.	
MMLRG (x_6_)	Medical security indicators	Higher MMLRG values may suggest issues in the hospital’s clinical or management processes, as death in such cases is often closely related to errors in the clinical process.	

CMI, case-mix index; ND, number of DRGs; TW, total weight; CEI, cost efficiency index; TEI, time efficiency index; MMLRG, mortality of middle and low-risk group cases.

### 2.3 Medical service performance evaluation procedures

#### 2.3.1 Data extraction and preparation

The data for this study were extracted from the SHDADSCP in the “Comprehensive Evaluation” section. All data were collected and organized in Excel format. The data preparation followed the following procedures:

1 Data extraction: The original data matrix was labeled as *X_ij_*, where *i* = 1, 2, …, *m* and *j* = 1, 2, …, *n*. Here, *m* represents the number of evaluation indicators, and *n* represents the number of study hospitals.
2 Data trends homogenization: The absolute negative indicators of CEI and TEI were homogenized using Equation 1. The relative negative indicator of MMLRG was homogenized using Equation 2 (30).

(1) $$X{'}_{\mathrm{ij}}=\frac{1}{X_{\mathrm{ij}}}$$

(2) $$X{'}_{\mathrm{ij}}=100-X_{\mathrm{ij}}$$

3 Data standardization: To eliminate the influence of varying dimensions, all data were standardized using Equation 3. This step ensured that each indicator contributed equally to the analysis, regardless of its original scale or unit of measurement.

(3) $$Z_{\mathrm{ij}}=\frac{X_{\mathrm{ij}}^{\prime }-{\bar{X}}_{\mathrm{ij}}^{\prime }}{S_{j}}$$

#### 2.3.2 EFA procedures

EFA, initially developed by Charles Spearman in 1904, is a multivariate statistical method. It classifies multiple variables into a few common factors based on the correlations among the variables. The fundamental concept involves decomposing original variables into two components: one is a linear combination of common factors (CFs) that condense most of the information in the original variables, and the other is a special factor that exhibits no correlation with the CFs. The main purpose is to explore the underlying structure beneath extensive observed data and identify latent factors influencing these data (31).

Following data standardization, we conducted EFA using SPSS 27.0 software. The main procedures were as follows:

1 Test data appropriateness. Before performing EFA, it is necessary to evaluate data appropriateness. Two commonly used metrics are the Kaiser-Meyer-Olkin (KMO) test and Bartlett’s test of sphericity. Data are deemed appropriate for EFA when the KMO value exceeds 0.6 or 0.7 and Bartlett’s test yields a significance level below 0.05.
2 Calculate communality values. Communality, denoted as $h_{i}^{2}$ , was calculated using Equation 4:

(4) $$h_{i}^{2}=\sum_{k=1}^{m}{a^{2}}_{\mathrm{ik}},$$

where i = 1,2,…,n.

3 Extract CFs. The principal component analysis (PCA) method was used to determine the number of CFs. Factors with accumulative variance contribution (AVC) ≥ 85% (32) were extracted. The AVC was calculated using Equation 5:

(5) $$\mathrm{AVC}=\sum \frac{\lambda_{i}}{\sum \lambda_{i}}\times 100\%,$$

where $\lambda_{i}$ represents the eigenvalues of each indicator.

4 Compute factor loading matrix. The factor loading matrix A was derived from the eigenvalues and their corresponding eigenvectors. The matrix was defined as Equation 6:

(6) $$A={\left(\sqrt{\lambda_{1}}\iota_{1}\sqrt{\lambda_{2}}\iota_{2}\cdots \sqrt{\lambda_{\mathrm{jj}}}\iota_{j}\right)}_{i\times j},$$

where λ represents the eigenvalue, ι represents the eigenvector, *i* = 1, 2, …, *m*; *j* = 1, 2, …, *n*; *m* represents the number of evaluation indicators, and *n* represents the number of study hospitals.

5 Rotate CFs. To enhance the interpretability of each CF, rotation was performed using the varimax method. The rotated factor loading matrix visually reflects the contribution of each variable to the principal components. A larger absolute value of a variable’s loading coefficient on a specific CF implies a stronger correlation between the variable and that factor.
6 Determine factor score functions. The factor score function $F_{k}$ was defined as Equation 7:

(7) $$F_{k}=\sum \omega_{\mathrm{ij}}z_{\mathrm{ij}},$$

where $\omega_{\mathrm{ij}}$ represents the coefficients of CF scores, $z_{\mathrm{ij}}$ represents the standardized data matrix, and *k* represents the number of CFs.

7 Calculate the evaluation scores for each study hospital. The comprehensive evaluation scores (CES) of each study hospital were calculated using Equation 8 and ranked accordingly:

(8) $$\mathrm{CES}=\sum (\frac{\mathrm{RVC}}{\mathrm{AVC}}\times F_{k}),$$

where RVC represents the rotated variance contribution.

### 2.4 HCA procedures

**Figure 2:**
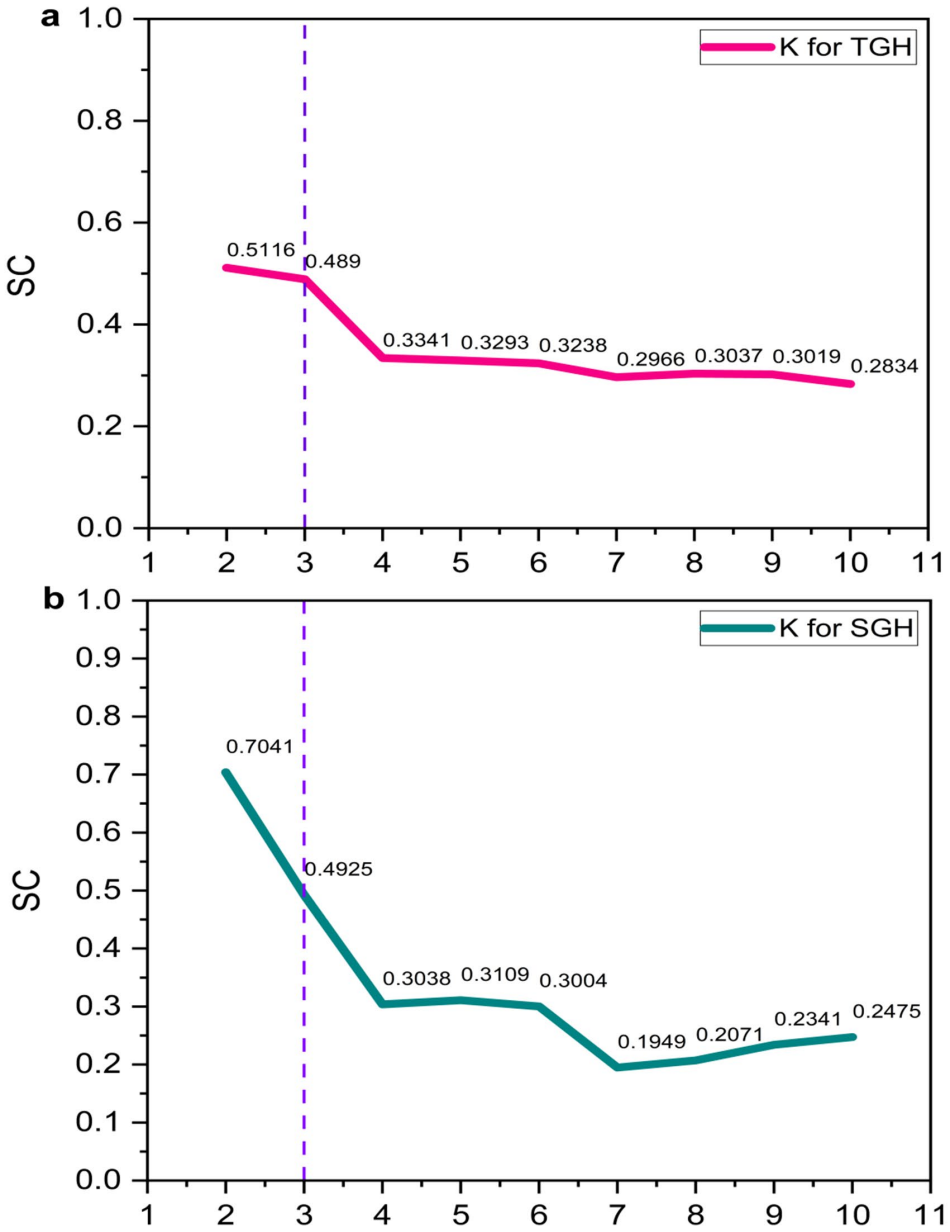
Determination of the optimal number of clusters using SC. TGH, tertiary general hospital; SGH, secondary general hospital; SC, Silhouette Coefficient. Panel **(a)** corresponds to the analysis of tertiary general hospitals (TGH), showing how the Silhouette Coefficient (SC) changes with the number of clusters (K). Panel **(b)** corresponds to the analysis of secondary general hospitals (SGH), illustrating the SC-K relationship for this hospital level.

HCA is a descriptive statistical method that groups original data into clusters by measuring distances between data points. The goal is to minimize intra-cluster heterogeneity and maximize inter-cluster heterogeneity. To account for inherent differences between hospital levels, SGHs and TGHs were clustered separately following these procedures:

1. Variable standardization. Prior to HCA, variables including CF_1_, CF_2_, CF_3_, and CES were standardized using the Z-score method.
2. Distance metric and clustering algorithm. The Squared Euclidean Distance was used to measure data point dissimilarity, and the Between-groups Linkage method served as the clustering algorithm.
3. Optimal cluster determination. The Silhouette Coefficient (SC) (33, 34) was initially used to identify the optimal number of clusters (K), theoretically set at the highest SC value. Professional interpretation was additionally incorporated to refine this determination. The final optimal number of clusters for HCA was three, as illustrated in Figure 2.
4. Cluster definition. Three clusters were defined by research members according to the average values of six evaluation indicators per cluster.

### 2.5 Statistical analysis

All statistical analyses were performed using SPSS 27.0. The normality of continuous variables was assessed using the Shapiro–Wilk test. Non-normally distributed variables were described using the median and interquartile range [M (IQR)]. To examine the characteristic disparities across clusters, the Kruskal-Wallis H test was applied to non-normally distributed variables. A *p*-value less than 0.05 was considered statistically significant.

## 3 Results

### 3.1 EFA results

The KMO test and Bartlett’s test of sphericity confirmed the data suitability for EFA, with a KMO value was 0.726 and significant Bartlett’s test results (*p* < 0.001). Three CFs were extracted according to the extraction principle. CF_1_ explained 48.502% of the total variance (rotated eigenvalue = 2.910), CF_2_ explained 19.052% (rotated eigenvalue = 1.143), and CF_3_ explained 18.085% (rotated eigenvalue = 1.085), collectively accounting for 85.639% of the cumulative variance. All communality values of the six indicators exceeded 0.790, with the largest in MMLRG (0.956), followed by TEI (0.918), CMI (0.874), ND (0.806), TW (0.794), and CEI (0.791), indicating strong representation of original indicators by the extracted CFs. Detailed variance contributions and eigenvalues are presented in Table 2.

**Table 2:** Eigenvalues of the correlation matrix and variance contribution rate.

Component	Initial eigenvalues			Rotation sums of squared loading		
	Eigenvalue	Variance contribution rate (%)	Cumulative variance contribution rate (%)	Eigenvalue	Variance contribution rate (%)	Cumulative variance contribution rate (%)
CF_1_	3.147	52.445	52.445	2.910	48.502	48.502
CF_2_	1.247	20.779	73.224	1.143	19.052	67.554
CF_3_	0.745	12.415	85.639	1.085	18.085	85.639
CF_4_	0.466	7.766	93.405			
CF_5_	0.254	4.241	97.646			
CF_6_	0.141	2.354	100.000			

CF, common factor.

The rotated factor loading matrix showed that CF_1_ had high loadings on CMI (0.903), ND (0.823), and TW (0.804), reflecting hospital medical abilities, thus labeled as the medical ability factor. CF_2_ exhibited a dominant loading on TEI (0.931), labeled as the medical efficiency factor, while CF_3_ demonstrated a high loading on MMLRG (0.964), designated as the medical security factor. Factor loading details are presented in Table 3.

**Table 3:** Factor loading matrix and rotated factor loading matrix.

Indicators	Factor loading matrix			Rotated factor loading matrix		
	CF_1_	CF_2_	CF_3_	CF_1_	CF_2_	CF_3_
CMI (x_1_)	0.894	−0.247	0.113	0.903	0.214	−0.113
ND (x_2_)	0.872	0.071	−0.200	0.823	0.159	0.323
TW (x_3_)	0.866	−0.056	0.200	0.804	0.382	−0.031
CEI (x_4_)	−0.768	0.345	0.287	−0.869	0.183	−0.054
TEI (x_5_)	0.434	0.646	0.559	0.127	0.931	0.188
MMLRG (x_6_)	0.239	0.801	−0.508	0.036	0.163	0.964

CMI, case-mix index; ND, number of DRGs; TW, total weight; CEI, cost efficiency index; TEI, time efficiency index; MMLRG, mortality of middle and low-risk group cases.

Common factor scores for each study hospital were calculated based on the factor score coefficient matrix (Table 4):


(9) $$\begin{array}{c} {\mathrm{CF}}_{1}=0.310\times {\mathrm{Zx}}_{1}+0.278\times {\mathrm{Zx}}_{2}+0.237\times {\mathrm{Zx}}_{3}\\ -0.369\times {\mathrm{Zx}}_{4}-0.136\times {\mathrm{Zx}}_{5}-0.043\times {\mathrm{Zx}}_{6} \end{array}$$

(10) $$\begin{array}{c} {\mathrm{CF}}_{2}=0.085\times {\mathrm{Zx}}_{1}-0.083\times {\mathrm{Zx}}_{2}+0.259\times {\mathrm{Zx}}_{3}\\ +0.377\times {\mathrm{Zx}}_{4}+0.908\times {\mathrm{Zx}}_{5}-0.110\times {\mathrm{Zx}}_{6} \end{array}$$

(11) $$\begin{array}{c} {\mathrm{CF}}_{3}=-0.200\times {\mathrm{Zx}}_{1}+0.261\times {\mathrm{Zx}}_{2}-0.163\times {\mathrm{Zx}}_{3}\\ -0.084\times {\mathrm{Zx}}_{4}-0.079\times {\mathrm{Zx}}_{5}+0.932\times {\mathrm{Zx}}_{6} \end{array}$$

**Table 4:** Factor score coefficient matrix.

Indicators	CF_1_	CF_2_	CF_3_
CMI (x_1_)	0.310	0.085	−0.200
ND (x_2_)	0.278	−0.083	0.261
TW (x_3_)	0.237	0.259	−0.163
CEI (x_4_)	−0.369	0.377	−0.084
TEI (x_5_)	−0.136	0.908	−0.079
MMLRG (x_6_)	−0.043	−0.110	0.932

CMI, case-mix index; ND, number of DRGs; TW, total weight; CEI, cost efficiency index; TEI, time efficiency index; MMLRG, mortality of middle and low-risk group cases.

The CES for each study hospital was computed using the following function:


(12) $$\mathrm{CES}=0.566\times {\mathrm{CF}}_{1}+0.222\times {\mathrm{CF}}_{2}+0.211\times {\mathrm{CF}}_{3}$$

Ranking based on CES showed all top 10 hospitals were TGHs, with four located in Chengdu and one each in Luzhou, Suining, Deyang, Mianyang, and Nanchong. Conversely, the bottom 10 hospitals were all SGHs, predominantly concentrated in northeastern Sichuan (Guangyuan, Dazhou, Guang’an, Nanchong, Bazhong), with seven of the 10 located there. Top and the bottom 10 hospital details are in Table 5, and full EFA rankings for 306 study hospitals are in Table 6.

**Table 5:** The information of the top 10 and the bottom 10 hospitals based on EFA.

Hospital code	Hospital level	Region	CMI	ND	TW	CEI	TEI	MMLRG	CF1	CF2	CF3	CES	Ranking
Top 10 hospitals													
Hospital 1	Tertiary	Chengdu	1.81	688	423,760.08	1.21	0.77	0.05	5.2025	4.3696	−2.2361	3.4428	1
Hospital 2	Tertiary	Chengdu	1.42	719	278,580.96	1.24	0.83	0.12	3.7840	2.5935	−1.1592	2.4729	2
Hospital 18	Tertiary	Luzhou	1.33	713	189,932.71	1.16	0.86	0.11	3.0554	1.7628	−0.6740	1.9785	3
Hospital 3	Tertiary	Chengdu	1.24	681	126,680.29	1.02	0.82	0.06	2.2673	1.8086	−0.3534	1.6102	4
Hospital 37	Tertiary	Suining	1.2	688	129,339.9	0.92	0.83	0.04	2.1174	1.8244	−0.2969	1.5408	5
Hospital 22	Tertiary	Deyang	1.19	698	109,574.31	0.93	0.83	0.02	2.0097	1.6686	−0.1576	1.4747	6
Hospital 4	Tertiary	Chengdu	1.44	590	77,231.31	1.19	0.9	0.1	2.4419	0.7886	−0.4956	1.4526	7
Hospital 26	Tertiary	Mianyang	1.16	689	127,757.07	0.88	0.85	0.07	2.0106	1.6718	−0.2777	1.4505	8
Hospital 41	Tertiary	Nanchong	1.09	701	146,486.33	1.04	0.98	0.07	2.3561	0.5564	−0.1210	1.4316	9
Hospital 48	Tertiary	Yibin	1.12	692	119,917.1	0.82	0.86	0.05	1.8156	1.6012	−0.1793	1.3453	10
Bottom 10 hospitals													
Hospital 193	Secondary	Ya’an	0.45	99	996	0.48	1.05	0	−1.9204	0.4069	0.1029	−0.9749	297
Hospital 257	Secondary	Guangyuan	0.64	118	702.37	0.69	1.49	1.25	−0.4948	−1.8251	−1.4455	−0.9902	298
Hospital 286	Secondary	Dazhou	0.72	246	4,097.06	0.72	2.13	1.88	0.1958	−3.0898	−2.0586	−1.0095	299
Hospital 149	Secondary	Panzhihua	0.63	232	5,289.82	0.6	1.79	1.24	−0.4178	−2.3381	−1.2436	−1.0179	300
Hospital 276	Secondary	Guang’an	0.72	227	1,873.11	0.61	1.22	3.22	−0.3793	−0.3583	−4.1284	−1.1653	301
Hospital 266	Secondary	Nanchong	0.81	265	3,854.96	0.51	1.19	3.89	−0.4662	0.2791	−5.1682	−1.2924	302
Hospital 297	Secondary	Bazhong	0.67	140	1,010.52	0.72	1.9	3.39	−0.0619	−2.4777	−4.2090	−1.4732	303
Hospital 239	Secondary	Zigong	0.48	28	30.16	0.37	3.11	0	−2.0697	−2.8943	0.1513	−1.7821	304
Hospital 164	Secondary	Guangyuan	0.85	98	1,987.64	1.07	2.29	7.05	1.0135	−2.8653	−9.2218	−2.0083	305
Hospital 269	Secondary	Nanchong	0.69	80	1,233.74	0.6	1.11	6.61	−0.5946	0.7846	−8.9117	−2.0427	306

**Table 6:** Overall ranking results of the study hospitals.

Hospital code	Hospital level	Region	CF1	CF2	CF3	CES	Ranking
Hospital 1	Tertiary	Chengdu	5.2025	4.3696	−2.2361	3.4428	1
Hospital 2	Tertiary	Chengdu	3.7840	2.5935	−1.1592	2.4729	2
Hospital 18	Tertiary	Luzhou	3.0554	1.7628	−0.6740	1.9785	3
Hospital 3	Tertiary	Chengdu	2.2673	1.8086	−0.3534	1.6102	4
Hospital 37	Tertiary	Suining	2.1174	1.8244	−0.2969	1.5408	5
Hospital 22	Tertiary	Deyang	2.0097	1.6686	−0.1576	1.4747	6
Hospital 4	Tertiary	Chengdu	2.4419	0.7886	−0.4956	1.4526	7
Hospital 26	Tertiary	Mianyang	2.0106	1.6718	−0.2777	1.4505	8
Hospital 41	Tertiary	Nanchong	2.3561	0.5564	−0.1210	1.4316	9
Hospital 48	Tertiary	Yibin	1.8156	1.6012	−0.1793	1.3453	10
Hospital 5	Tertiary	Chengdu	1.9873	0.8717	0.0607	1.3311	11
Hospital 42	Tertiary	Nanchong	2.1681	0.4517	−0.0441	1.3181	12
Hospital 53	Tertiary	Dazhou	1.7000	1.0770	0.0022	1.2018	13
Hospital 6	Tertiary	Chengdu	1.7160	0.9065	0.0702	1.1873	14
Hospital 27	Tertiary	Mianyang	1.7591	0.7941	0.0312	1.1785	15
Hospital 40	Tertiary	Leshan	1.7413	0.8727	−0.0792	1.1626	16
Hospital 11	Tertiary	Zigong	1.6222	0.7046	0.1997	1.1167	17
Hospital 51	Tertiary	Guang’an	1.3831	1.3595	−0.0505	1.0740	18
Hospital 49	Tertiary	Yibin	1.6030	0.6001	0.1010	1.0619	19
Hospital 16	Tertiary	Panzhihua	1.5905	0.7828	−0.0694	1.0594	20
Hospital 7	Tertiary	Chengdu	1.5651	0.5220	−0.0059	1.0005	21
Hospital 12	Tertiary	Zigong	1.5920	0.1063	0.3254	0.9933	22
Hospital 8	Tertiary	Chengdu	1.4497	0.4852	0.1427	0.9584	23
Hospital 9	Tertiary	Chengdu	1.6433	−0.2235	0.3435	0.9530	24
Hospital 28	Tertiary	Mianyang	1.4385	0.3788	0.2060	0.9417	25
Hospital 46	Tertiary	Meishan	1.2869	0.8509	0.1141	0.9413	26
Hospital 35	Tertiary	Guangyuan	1.2974	0.5743	0.2547	0.9156	27
Hospital 10	Tertiary	Chengdu	1.4484	0.0666	0.2405	0.8853	28
Hospital 57	Tertiary	Ya’an	1.2960	0.4289	0.2068	0.8724	29
Hospital 65	Tertiary	Liangshan	1.5367	−0.5547	0.3613	0.8229	30
Hospital 58	Tertiary	Bazhong	1.3280	−0.1597	0.1977	0.7579	31
Hospital 23	Tertiary	Deyang	1.0580	0.4889	0.2390	0.7578	32
Hospital 61	Tertiary	Ziyang	1.1637	−0.1899	0.4482	0.7111	33
Hospital 38	Tertiary	Neijiang	1.1945	−0.3369	0.4887	0.7044	34
Hospital 36	Tertiary	Guangyuan	1.1283	−0.1710	0.3222	0.6686	35
Hospital 29	Tertiary	Mianyang	0.9653	0.3274	0.1905	0.6592	36
Hospital 47	Tertiary	Meishan	0.6633	1.0855	0.2012	0.6588	37
Hospital 43	Tertiary	Nanchong	1.0970	−0.2405	0.3655	0.6447	38
Hospital 20	Tertiary	Luzhou	1.1149	−0.5830	0.5830	0.6246	39
Hospital 66	Tertiary	Liangshan	1.1909	−0.7596	0.4427	0.5988	40
Hospital 68	Tertiary	Chengdu	0.9853	−0.3271	0.5067	0.5920	41
Hospital 54	Tertiary	Dazhou	0.8294	0.1485	0.4213	0.5913	42
Hospital 94	Tertiary	Suining	0.8878	−0.0808	0.4837	0.5866	43
Hospital 24	Tertiary	Deyang	0.8616	−0.0087	0.3918	0.5684	44
Hospital 25	Tertiary	Deyang	0.7622	0.2736	0.3462	0.5652	45
Hospital 14	Tertiary	Zigong	0.8102	0.1354	0.3357	0.5595	46
Hospital 15	Tertiary	Zigong	1.0301	−0.4885	0.3885	0.5566	47
Hospital 117	Tertiary	Guang’an	0.4634	0.9240	0.3497	0.5412	48
Hospital 55	Tertiary	Dazhou	0.8162	−0.0432	0.4068	0.5382	49
Hospital 52	Tertiary	Guang’an	0.8572	−0.0187	0.1867	0.5204	50
Hospital 13	Tertiary	Zigong	0.6028	0.4402	0.3660	0.5161	51
Hospital 30	Tertiary	Mianyang	0.9403	−0.5707	0.4562	0.5018	52
Hospital 39	Tertiary	Neijiang	0.8367	−0.2153	0.3559	0.5009	53
Hospital 120	Tertiary	Dazhou	0.8648	−0.0476	0.0733	0.4944	54
Hospital 21	Tertiary	Luzhou	0.5045	0.4485	0.5166	0.4941	55
Hospital 69	Tertiary	Chengdu	0.6442	0.2774	0.2958	0.4886	56
Hospital 45	Tertiary	Nanchong	1.1158	−0.9069	0.2005	0.4725	57
Hospital 31	Tertiary	Mianyang	0.9674	−0.7646	0.4433	0.4713	58
Hospital 19	Tertiary	Luzhou	0.5228	0.2613	0.5336	0.4665	59
Hospital 126	Tertiary	Ziyang	0.9267	−0.7764	0.5294	0.4639	60
Hospital 80	Tertiary	Luzhou	0.6578	0.0475	0.3701	0.4610	61
Hospital 124	Tertiary	Bazhong	0.4141	0.6332	0.3767	0.4544	62
Hospital 97	Tertiary	Neijiang	0.6146	−0.0572	0.5212	0.4452	63
Hospital 44	Tertiary	Nanchong	0.9641	−0.7641	0.3115	0.4418	64
Hospital 62	Tertiary	Ziyang	0.8275	−0.4423	0.3390	0.4417	65
Hospital 17	Tertiary	Panzhihua	0.8712	−0.7515	0.5053	0.4329	66
Hospital 56	Tertiary	Dazhou	0.5786	−0.0264	0.5081	0.4288	67
Hospital 33	Tertiary	Mianyang	1.0514	−1.1130	0.2887	0.4089	68
Hospital 119	Tertiary	Guang’an	0.6779	−0.3762	0.5098	0.4077	69
Hospital 59	Tertiary	Bazhong	0.5452	0.2013	0.2367	0.4032	70
Hospital 84	Tertiary	Deyang	0.5354	−0.0386	0.4522	0.3899	71
Hospital 83	Tertiary	Deyang	0.6055	0.0667	0.1286	0.3846	72
Hospital 81	Tertiary	Luzhou	0.2271	0.6084	0.5732	0.3845	73
Hospital 127	Tertiary	Ziyang	0.5313	0.3602	0.0080	0.3823	74
Hospital 71	Tertiary	Chengdu	0.4352	0.4102	0.1585	0.3709	76
Hospital 32	Tertiary	Mianyang	0.9569	−1.2150	0.4245	0.3614	77
Hospital 118	Tertiary	Guang’an	0.3962	0.3433	0.2845	0.3605	78
Hospital 104	Tertiary	Nanchong	0.7022	−0.7977	0.6360	0.3545	79
Hospital 74	Tertiary	Chengdu	0.9750	−1.2234	0.3122	0.3461	80
Hospital 72	Tertiary	Chengdu	0.5346	−0.2246	0.3836	0.3337	81
Hospital 64	Tertiary	Ganzi	0.8441	−1.1279	0.4408	0.3204	82
Hospital 75	Tertiary	Chengdu	0.5211	−0.5415	0.6081	0.3030	83
Hospital 50	Tertiary	Yibin	0.5234	−0.4837	0.4788	0.2899	84
Hospital 70	Tertiary	Chengdu	0.5260	−0.5013	0.4804	0.2878	85
Hospital 76	Tertiary	Chengdu	0.3163	−0.0302	0.5246	0.2830	87
Hospital 73	Tertiary	Chengdu	0.5268	−0.5725	0.4648	0.2692	88
Hospital 91	Tertiary	Guangyuan	0.4112	0.1785	−0.0583	0.2601	89
Hospital 95	Tertiary	Suining	0.3398	−0.0030	0.3228	0.2598	90
Hospital 86	Tertiary	Mianyang	0.2291	0.2919	0.2885	0.2553	91
Hospital 105	Tertiary	Nanchong	0.4405	−0.3630	0.3562	0.2439	92
Hospital 87	Tertiary	Mianyang	0.2223	0.3909	0.1103	0.2359	93
Hospital 85	Tertiary	Deyang	0.2786	−0.0177	0.3431	0.2262	95
Hospital 60	Tertiary	Bazhong	0.6400	−1.1535	0.4939	0.2104	96
Hospital 67	Tertiary	Liangshan	0.5441	−1.0169	0.5979	0.2084	97
Hospital 106	Tertiary	Nanchong	0.2483	0.2070	0.0411	0.1952	100
Hospital 101	Tertiary	Leshan	0.3111	0.1159	−0.0792	0.1851	102
Hospital 90	Tertiary	Guangyuan	0.2543	−0.1705	0.3255	0.1747	103
Hospital 98	Tertiary	Neijiang	0.5158	−1.1837	0.6580	0.1680	104
Hospital 99	Tertiary	Neijiang	0.3324	−0.2876	0.0311	0.1309	108
Hospital 92	Tertiary	Guangyuan	0.3758	−0.7538	0.3170	0.1123	109
Hospital 89	Tertiary	Mianyang	0.2225	−0.1415	0.0819	0.1118	110
Hospital 63	Tertiary	Aba	0.5281	−1.2546	0.3925	0.1032	113
Hospital 103	Tertiary	Leshan	0.2443	−0.5446	0.3643	0.0943	114
Hospital 102	Tertiary	Leshan	0.4995	−1.0967	0.2596	0.0940	115
Hospital 109	Tertiary	Yibin	−0.0216	−0.0200	0.5201	0.0931	116
Hospital 133	Tertiary	Liangshan	−0.2121	0.3594	0.5978	0.0859	119
Hospital 34	Tertiary	Mianyang	0.1786	−0.3189	0.2568	0.0845	120
Hospital 77	Tertiary	Chengdu	0.4888	−0.9297	0.0337	0.0774	121
Hospital 130	Tertiary	Liangshan	−0.2303	0.4229	0.4581	0.0602	123
Hospital 93	Tertiary	Guangyuan	0.7241	−1.9739	0.3920	0.0543	125
Hospital 88	Tertiary	Mianyang	0.1172	−0.4169	0.3476	0.0471	126
Hospital 108	Tertiary	Yibin	−0.2481	0.4474	0.3717	0.0374	129
Hospital 131	Tertiary	Liangshan	−0.1673	0.0148	0.5773	0.0304	133
Hospital 129	Tertiary	Liangshan	−0.3384	0.5382	0.4357	0.0199	135
Hospital 107	Tertiary	Nanchong	0.3526	−0.2634	−0.5810	0.0185	136
Hospital 78	Tertiary	Panzhihua	−0.0231	−0.3960	0.5481	0.0147	139
Hospital 113	Tertiary	Yibin	−0.2005	0.0578	0.4753	−0.0004	142
Hospital 96	Tertiary	Suining	0.0077	−0.1348	0.0477	−0.0155	146
Hospital 112	Tertiary	Yibin	−0.1166	−0.0716	0.2639	−0.0262	148
Hospital 100	Tertiary	Neijiang	0.2177	−1.2054	0.5585	−0.0265	149
Hospital 123	Tertiary	Ya’an	0.1381	−0.8863	0.3621	−0.0422	150
Hospital 111	Tertiary	Yibin	−0.4765	0.6552	0.3737	−0.0454	151
Hospital 121	Tertiary	Ya’an	−0.1621	−0.2928	0.4659	−0.0585	155
Hospital 79	Tertiary	Panzhihua	0.1494	−0.7269	0.0797	−0.0600	156
Hospital 122	Tertiary	Ya’an	−0.2647	−0.2105	0.5731	−0.0756	161
Hospital 114	Tertiary	Yibin	−0.7078	1.0011	0.4330	−0.0870	164
Hospital 115	Tertiary	Yibin	−0.3386	−0.1214	0.5005	−0.1130	168
Hospital 132	Tertiary	Liangshan	−0.0733	−0.9754	0.6648	−0.1178	169
Hospital 110	Tertiary	Yibin	−0.3006	−0.2204	0.4384	−0.1266	170
Hospital 82	Tertiary	Luzhou	0.0150	−0.8756	−0.1161	−0.2104	183
Hospital 125	Tertiary	Bazhong	−0.2643	0.0982	−0.5099	−0.2354	189
Hospital 128	Tertiary	Aba	−0.2540	−0.9550	0.5669	−0.2361	190
Hospital 116	Tertiary	Yibin	0.1580	−2.7967	0.2745	−0.4735	243

### 3.2 HCA results

#### 3.2.1 HCA for TGHs

Three distinct clusters were identified among TGHs: the “Excellent” cluster (Cluster 1, *n* = 41, 30.83%), “Middle” cluster (Cluster 2, *n* = 76, 57.14%), and “Inferior” cluster (Cluster 3, *n* = 16, 12.03%). Performance metrics showed significant inter-cluster differences: (1) the “Excellent” cluster outperformed “Middle” and “Inferior” clusters in CMI (1.02 vs. 0.80 vs. 0.68, *p* < 0.001), ND (663.00 vs. 575.00 vs. 517.50, *p* < 0.001), TW (77,231.31 vs. 28,130.14 vs. 19,023.35, *p* < 0.001), and TEI (0.92 vs. 1.06 vs. 1.02, *p* < 0.001); (2) the “Inferior” cluster demonstrated the best CEI (0.61 vs. 0.82 vs. 0.85, *p* < 0.001) and MMLRG (0.05 vs. 0.09 vs. 0.09, *p* = 0.011). Detailed clustering results and inter-cluster comparisons are presented in Tables 7, 8.

**Table 7:** Results of HCA for TGHs.

Hospital code	Hospital level	Region	Cluster	Cluster definition
Hospital 1	Tertiary	Chengdu	1	Excellent
Hospital 2	Tertiary	Chengdu	1	Excellent
Hospital 18	Tertiary	Luzhou	1	Excellent
Hospital 3	Tertiary	Chengdu	1	Excellent
Hospital 37	Tertiary	Suining	1	Excellent
Hospital 22	Tertiary	Deyang	1	Excellent
Hospital 4	Tertiary	Chengdu	1	Excellent
Hospital 26	Tertiary	Mianyang	1	Excellent
Hospital 41	Tertiary	Nanchong	1	Excellent
Hospital 48	Tertiary	Yibin	1	Excellent
Hospital 5	Tertiary	Chengdu	1	Excellent
Hospital 42	Tertiary	Nanchong	1	Excellent
Hospital 53	Tertiary	Dazhou	1	Excellent
Hospital 6	Tertiary	Chengdu	1	Excellent
Hospital 27	Tertiary	Mianyang	1	Excellent
Hospital 40	Tertiary	Leshan	1	Excellent
Hospital 11	Tertiary	Zigong	1	Excellent
Hospital 51	Tertiary	Guang’an	1	Excellent
Hospital 49	Tertiary	Yibin	1	Excellent
Hospital 16	Tertiary	Panzhihua	1	Excellent
Hospital 7	Tertiary	Chengdu	1	Excellent
Hospital 12	Tertiary	Zigong	2	Middle
Hospital 8	Tertiary	Chengdu	1	Excellent
Hospital 9	Tertiary	Chengdu	2	Middle
Hospital 28	Tertiary	Mianyang	1	Excellent
Hospital 46	Tertiary	Meishan	1	Excellent
Hospital 35	Tertiary	Guangyuan	1	Excellent
Hospital 10	Tertiary	Chengdu	2	Middle
Hospital 57	Tertiary	Ya’an	1	Excellent
Hospital 65	Tertiary	Liangshan	2	Middle
Hospital 58	Tertiary	Bazhong	2	Middle
Hospital 23	Tertiary	Deyang	1	Excellent
Hospital 61	Tertiary	Ziyang	2	Middle
Hospital 38	Tertiary	Neijiang	2	Middle
Hospital 36	Tertiary	Guangyuan	2	Middle
Hospital 29	Tertiary	Mianyang	1	Excellent
Hospital 47	Tertiary	Meishan	1	Excellent
Hospital 43	Tertiary	Nanchong	2	Middle
Hospital 20	Tertiary	Luzhou	2	Middle
Hospital 66	Tertiary	Liangshan	2	Middle
Hospital 68	Tertiary	Chengdu	2	Middle
Hospital 54	Tertiary	Dazhou	2	Middle
Hospital 94	Tertiary	Suining	2	Middle
Hospital 24	Tertiary	Deyang	2	Middle
Hospital 25	Tertiary	Deyang	2	Middle
Hospital 14	Tertiary	Zigong	2	Middle
Hospital 15	Tertiary	Zigong	2	Middle
Hospital 117	Tertiary	Guang’an	1	Excellent
Hospital 55	Tertiary	Dazhou	2	Middle
Hospital 52	Tertiary	Guang’an	2	Middle
Hospital 13	Tertiary	Zigong	1	Excellent
Hospital 30	Tertiary	Mianyang	2	Middle
Hospital 39	Tertiary	Neijiang	2	Middle
Hospital 120	Tertiary	Dazhou	2	Middle
Hospital 21	Tertiary	Luzhou	2	Middle
Hospital 69	Tertiary	Chengdu	2	Middle
Hospital 45	Tertiary	Nanchong	2	Middle
Hospital 31	Tertiary	Mianyang	2	Middle
Hospital 19	Tertiary	Luzhou	2	Middle
Hospital 126	Tertiary	Ziyang	2	Middle
Hospital 80	Tertiary	Luzhou	2	Middle
Hospital 124	Tertiary	Bazhong	1	Excellent
Hospital 97	Tertiary	Neijiang	2	Middle
Hospital 44	Tertiary	Nanchong	2	Middle
Hospital 62	Tertiary	Ziyang	2	Middle
Hospital 17	Tertiary	Panzhihua	2	Middle
Hospital 56	Tertiary	Dazhou	2	Middle
Hospital 33	Tertiary	Mianyang	2	Middle
Hospital 119	Tertiary	Guang’an	2	Middle
Hospital 59	Tertiary	Bazhong	2	Middle
Hospital 84	Tertiary	Deyang	2	Middle
Hospital 83	Tertiary	Deyang	2	Middle
Hospital 81	Tertiary	Luzhou	3	Inferior
Hospital 127	Tertiary	Ziyang	1	Excellent
Hospital 71	Tertiary	Chengdu	1	Excellent
Hospital 32	Tertiary	Mianyang	2	Middle
Hospital 118	Tertiary	Guang’an	1	Excellent
Hospital 104	Tertiary	Nanchong	2	Middle
Hospital 74	Tertiary	Chengdu	2	Middle
Hospital 72	Tertiary	Chengdu	2	Middle
Hospital 64	Tertiary	Ganzi	2	Middle
Hospital 75	Tertiary	Chengdu	2	Middle
Hospital 50	Tertiary	Yibin	2	Middle
Hospital 70	Tertiary	Chengdu	2	Middle
Hospital 76	Tertiary	Chengdu	2	Middle
Hospital 73	Tertiary	Chengdu	2	Middle
Hospital 91	Tertiary	Guangyuan	1	Excellent
Hospital 95	Tertiary	Suining	2	Middle
Hospital 86	Tertiary	Mianyang	3	Inferior
Hospital 105	Tertiary	Nanchong	2	Middle
Hospital 87	Tertiary	Mianyang	1	Excellent
Hospital 85	Tertiary	Deyang	2	Middle
Hospital 60	Tertiary	Bazhong	2	Middle
Hospital 67	Tertiary	Liangshan	2	Middle
Hospital 106	Tertiary	Nanchong	1	Excellent
Hospital 101	Tertiary	Leshan	1	Excellent
Hospital 90	Tertiary	Guangyuan	2	Middle
Hospital 98	Tertiary	Neijiang	2	Middle
Hospital 99	Tertiary	Neijiang	2	Middle
Hospital 92	Tertiary	Guangyuan	2	Middle
Hospital 89	Tertiary	Mianyang	2	Middle
Hospital 63	Tertiary	Aba	2	Middle
Hospital 103	Tertiary	Leshan	2	Middle
Hospital 102	Tertiary	Leshan	2	Middle
Hospital 109	Tertiary	Yibin	3	Inferior
Hospital 133	Tertiary	Liangshan	3	Inferior
Hospital 34	Tertiary	Mianyang	2	Middle
Hospital 77	Tertiary	Chengdu	2	Middle
Hospital 130	Tertiary	Liangshan	3	Inferior
Hospital 93	Tertiary	Guangyuan	2	Middle
Hospital 88	Tertiary	Mianyang	2	Middle
Hospital 108	Tertiary	Yibin	3	Inferior
Hospital 131	Tertiary	Liangshan	3	Inferior
Hospital 129	Tertiary	Liangshan	3	Inferior
Hospital 107	Tertiary	Nanchong	1	Excellent
Hospital 78	Tertiary	Panzhihua	2	Middle
Hospital 113	Tertiary	Yibin	3	Inferior
Hospital 96	Tertiary	Suining	2	Middle
Hospital 112	Tertiary	Yibin	3	Inferior
Hospital 100	Tertiary	Neijiang	2	Middle
Hospital 123	Tertiary	Ya’an	2	Middle
Hospital 111	Tertiary	Yibin	3	Inferior
Hospital 121	Tertiary	Ya’an	3	Inferior
Hospital 79	Tertiary	Panzhihua	2	Middle
Hospital 122	Tertiary	Ya’an	3	Inferior
Hospital 114	Tertiary	Yibin	3	Inferior
Hospital 115	Tertiary	Yibin	3	Inferior
Hospital 132	Tertiary	Liangshan	2	Middle
Hospital 110	Tertiary	Yibin	3	Inferior
Hospital 82	Tertiary	Luzhou	2	Middle
Hospital 125	Tertiary	Bazhong	1	Excellent
Hospital 128	Tertiary	Aba	2	Middle
Hospital 116	Tertiary	Yibin	2	Middle

**Table 8:** Comparison analysis among different clusters of TGHs.

Indicators	Excellent median (IQR)	Middle median (IQR)	Inferior median (IQR)	*p*^a^
CMI	1.02 (0.83, 1.10)	0.80 (0.76, 0.88)	0.68 (0.64, 0.71)	<0.001
ND	663.00 (590.00, 680.00)	575.00 (520.75, 612.75)	517.50 (486.25, 533.00)	<0.001
TW	77,231.31 (33,848.48, 113,706.55)	28,130.14 (18,505.71, 39,746.17)	19,023.35 (15,368.54, 21,079.08)	<0.001
CEI	0.85 (0.76, 0.97)	0.82 (0.76, 0.87)	0.61 (0.55, 0.66)	<0.001
TEI	0.92 (0.88, 0.96)	1.06 (1.01, 1.12)	1.02 (0.96, 1.06)	<0.001
MMLRG	0.09 (0.05, 0.13)	0.09 (0.04, 0.18)	0.05 (0.00, 0.07)	0.011

a Based on the independent-sample Kruskal-Wallis test.

CMI, case-mix index; ND, number of DRGs; TW, total weight; CEI, cost efficiency index; TEI, time efficiency index; MMLRG, mortality of middle and low-risk groups cases.

#### 3.2.2 HCA for SGHs

SGHs were categorized into three clusters: “Excellent” (Cluster 1, *n* = 45, 26.01%), “Middle” (Cluster 2, *n* = 121, 69.94%), and “Inferior” (Cluster 3, *n* = 7, 4.05%). Significant inter-cluster differences were identified: (1) the “Excellent” cluster outperformed in ND (392.00 vs. 293.00 vs. 186.00, *p* < 0.001) and TW (7,088.34 vs. 4,072.28 vs. 1,873.11, *p* = 0.001); (2) the “Inferior” cluster demonstrated the highest CMI (0.81 vs. 0.70 vs. 0.64, *p* < 0.001) but the worst MMLRG (3.39 vs. 0.18 vs. 0.04, *p* < 0.001); (3) the “Middle” cluster exhibited optimal CEI (0.57 vs. 0.72 vs. 0.64, *p* < 0.001) and TEI (0.99 vs. 1.16 vs. 1.19, *p* < 0.001). Clustering results and comparative analyses are provided in Tables 9, 10.

**Table 9:** Results of HCA for SGHs.

Hospital code	Hospital level	Region	Cluster	Cluster definition
Hospital 134	Secondary	Chengdu	1	Excellent
Hospital 184	Secondary	Dazhou	2	Middle
Hospital 186	Secondary	Dazhou	2	Middle
Hospital 138	Secondary	Chengdu	1	Excellent
Hospital 260	Secondary	Nanchong	2	Middle
Hospital 218	Secondary	Liangshan	1	Excellent
Hospital 175	Secondary	Nanchong	2	Middle
Hospital 139	Secondary	Chengdu	1	Excellent
Hospital 136	Secondary	Chengdu	1	Excellent
Hospital 241	Secondary	Luzhou	2	Middle
Hospital 151	Secondary	Luzhou	2	Middle
Hospital 150	Secondary	Luzhou	2	Middle
Hospital 292	Secondary	Bazhong	2	Middle
Hospital 142	Secondary	Chengdu	1	Excellent
Hospital 176	Secondary	Nanchong	1	Excellent
Hospital 185	Secondary	Dazhou	2	Middle
Hospital 145	Secondary	Zigong	2	Middle
Hospital 152	Secondary	Luzhou	2	Middle
Hospital 230	Secondary	Chengdu	2	Middle
Hospital 226	Secondary	Chengdu	1	Excellent
Hospital 135	Secondary	Chengdu	1	Excellent
Hospital 277	Secondary	Dazhou	1	Excellent
Hospital 153	Secondary	Luzhou	2	Middle
Hospital 146	Secondary	Panzhihua	1	Excellent
Hospital 279	Secondary	Dazhou	2	Middle
Hospital 141	Secondary	Chengdu	1	Excellent
Hospital 165	Secondary	Neijiang	3	Inferior
Hospital 144	Secondary	Zigong	1	Excellent
Hospital 167	Secondary	Leshan	1	Excellent
Hospital 137	Secondary	Chengdu	1	Excellent
Hospital 228	Secondary	Chengdu	2	Middle
Hospital 187	Secondary	Dazhou	2	Middle
Hospital 217	Secondary	Liangshan	2	Middle
Hospital 168	Secondary	Leshan	1	Excellent
Hospital 169	Secondary	Leshan	2	Middle
Hospital 155	Secondary	Deyang	1	Excellent
Hospital 278	Secondary	Dazhou	2	Middle
Hospital 143	Secondary	Chengdu	1	Excellent
Hospital 298	Secondary	Ziyang	2	Middle
Hospital 271	Secondary	Guang’an	2	Middle
Hospital 273	Secondary	Guang’an	2	Middle
Hospital 227	Secondary	Chengdu	1	Excellent
Hospital 179	Secondary	Meishan	2	Middle
Hospital 140	Secondary	Chengdu	1	Excellent
Hospital 274	Secondary	Guang’an	2	Middle
Hospital 194	Secondary	Bazhong	2	Middle
Hospital 287	Secondary	Ya’an	2	Middle
Hospital 216	Secondary	Liangshan	2	Middle
Hospital 170	Secondary	Leshan	2	Middle
Hospital 177	Secondary	Nanchong	1	Excellent
Hospital 232	Secondary	Chengdu	1	Excellent
Hospital 242	Secondary	Luzhou	2	Middle
Hospital 243	Secondary	Luzhou	2	Middle
Hospital 272	Secondary	Guang’an	2	Middle
Hospital 159	Secondary	Mianyang	2	Middle
Hospital 156	Secondary	Mianyang	2	Middle
Hospital 189	Secondary	Ya’an	2	Middle
Hospital 233	Secondary	Chengdu	1	Excellent
Hospital 158	Secondary	Mianyang	1	Excellent
Hospital 220	Secondary	Liangshan	2	Middle
Hospital 181	Secondary	Meishan	2	Middle
Hospital 251	Secondary	Guangyuan	2	Middle
Hospital 229	Secondary	Chengdu	1	Excellent
Hospital 154	Secondary	Deyang	1	Excellent
Hospital 252	Secondary	Guangyuan	2	Middle
Hospital 234	Secondary	Chengdu	1	Excellent
Hospital 161	Secondary	Guangyuan	2	Middle
Hospital 224	Secondary	Liangshan	2	Middle
Hospital 219	Secondary	Liangshan	1	Excellent
Hospital 231	Secondary	Chengdu	2	Middle
Hospital 163	Secondary	Guangyuan	2	Middle
Hospital 162	Secondary	Guangyuan	1	Excellent
Hospital 222	Secondary	Liangshan	2	Middle
Hospital 235	Secondary	Chengdu	2	Middle
Hospital 147	Secondary	Panzhihua	2	Middle
Hospital 195	Secondary	Ziyang	1	Excellent
Hospital 221	Secondary	Liangshan	2	Middle
Hospital 182	Secondary	Yibin	2	Middle
Hospital 261	Secondary	Nanchong	2	Middle
Hospital 236	Secondary	Zigong	2	Middle
Hospital 188	Secondary	Ya’an	2	Middle
Hospital 180	Secondary	Meishan	2	Middle
Hospital 223	Secondary	Liangshan	2	Middle
Hospital 157	Secondary	Mianyang	2	Middle
Hospital 246	Secondary	Deyang	1	Excellent
Hospital 299	Secondary	Aba	2	Middle
Hospital 183	Secondary	Guang’an	2	Middle
Hospital 301	Secondary	Liangshan	2	Middle
Hospital 192	Secondary	Ya’an	2	Middle
Hospital 253	Secondary	Guangyuan	2	Middle
Hospital 166	Secondary	Neijiang	1	Excellent
Hospital 263	Secondary	Nanchong	2	Middle
Hospital 281	Secondary	Dazhou	2	Middle
Hospital 288	Secondary	Ya’an	2	Middle
Hospital 293	Secondary	Bazhong	2	Middle
Hospital 255	Secondary	Guangyuan	2	Middle
Hospital 160	Secondary	Mianyang	2	Middle
Hospital 191	Secondary	Ya’an	2	Middle
Hospital 264	Secondary	Nanchong	2	Middle
Hospital 172	Secondary	Leshan	1	Excellent
Hospital 302	Secondary	Liangshan	2	Middle
Hospital 303	Secondary	Liangshan	2	Middle
Hospital 196	Secondary	Aba	2	Middle
Hospital 178	Secondary	Nanchong	2	Middle
Hospital 284	Secondary	Dazhou	2	Middle
Hospital 294	Secondary	Bazhong	2	Middle
Hospital 171	Secondary	Leshan	2	Middle
Hospital 256	Secondary	Guangyuan	2	Middle
Hospital 211	Secondary	Ganzi	2	Middle
Hospital 237	Secondary	Zigong	2	Middle
Hospital 208	Secondary	Ganzi	2	Middle
Hospital 206	Secondary	Ganzi	2	Middle
Hospital 304	Secondary	Liangshan	2	Middle
Hospital 268	Secondary	Nanchong	2	Middle
Hospital 262	Secondary	Nanchong	2	Middle
Hospital 190	Secondary	Ya’an	2	Middle
Hospital 215	Secondary	Ganzi	2	Middle
Hospital 245	Secondary	Deyang	2	Middle
Hospital 197	Secondary	Aba	2	Middle
Hospital 282	Secondary	Dazhou	1	Excellent
Hospital 248	Secondary	Mianyang	2	Middle
Hospital 285	Secondary	Dazhou	2	Middle
Hospital 244	Secondary	Luzhou	1	Excellent
Hospital 289	Secondary	Ya’an	2	Middle
Hospital 199	Secondary	Aba	2	Middle
Hospital 259	Secondary	Neijiang	2	Middle
Hospital 283	Secondary	Dazhou	2	Middle
Hospital 200	Secondary	Aba	2	Middle
Hospital 290	Secondary	Ya’an	2	Middle
Hospital 212	Secondary	Ganzi	2	Middle
Hospital 198	Secondary	Aba	2	Middle
Hospital 202	Secondary	Aba	2	Middle
Hospital 225	Secondary	Liangshan	2	Middle
Hospital 210	Secondary	Ganzi	2	Middle
Hospital 254	Secondary	Guangyuan	1	Excellent
Hospital 280	Secondary	Dazhou	1	Excellent
Hospital 295	Secondary	Bazhong	2	Middle
Hospital 148	Secondary	Panzhihua	2	Middle
Hospital 291	Secondary	Ya’an	1	Excellent
Hospital 214	Secondary	Ganzi	2	Middle
Hospital 265	Secondary	Nanchong	2	Middle
Hospital 213	Secondary	Ganzi	2	Middle
Hospital 270	Secondary	Yibin	3	Inferior
Hospital 201	Secondary	Aba	2	Middle
Hospital 204	Secondary	Aba	2	Middle
Hospital 207	Secondary	Ganzi	2	Middle
Hospital 247	Secondary	Mianyang	2	Middle
Hospital 203	Secondary	Aba	2	Middle
Hospital 267	Secondary	Nanchong	2	Middle
Hospital 275	Secondary	Guang’an	2	Middle
Hospital 300	Secondary	Ganzi	2	Middle
Hospital 305	Secondary	Liangshan	2	Middle
Hospital 306	Secondary	Liangshan	2	Middle
Hospital 296	Secondary	Bazhong	1	Excellent
Hospital 250	Secondary	Mianyang	2	Middle
Hospital 249	Secondary	Mianyang	1	Excellent
Hospital 205	Secondary	Aba	2	Middle
Hospital 173	Secondary	Leshan	2	Middle
Hospital 174	Secondary	Leshan	2	Middle
Hospital 240	Secondary	Panzhihua	2	Middle
Hospital 238	Secondary	Zigong	1	Excellent
Hospital 209	Secondary	Ganzi	2	Middle
Hospital 258	Secondary	Suining	2	Middle
Hospital 193	Secondary	Ya’an	2	Middle
Hospital 257	Secondary	Guangyuan	1	Excellent
Hospital 286	Secondary	Dazhou	1	Excellent
Hospital 149	Secondary	Panzhihua	1	Excellent
Hospital 276	Secondary	Guang’an	3	Inferior
Hospital 266	Secondary	Nanchong	3	Inferior
Hospital 297	Secondary	Bazhong	3	Inferior
Hospital 239	Secondary	Zigong	1	Excellent
Hospital 164	Secondary	Guangyuan	3	Inferior
Hospital 269	Secondary	Nanchong	3	Inferior

**Table 10:** Comparison analysis among different clusters of SGHs.

Indicators	Excellent median (IQR)	Middle median (IQR)	Inferior median (IQR)	*p*^a^
CMI	0.70 (0.65, 0.76)	0.64 (0.58, 0.70)	0.81 (0.69, 0.85)	<0.001
ND	392.00 (291.50, 437.50)	293.00 (245.50, 391.50)	186.00 (98.00, 265.00)	0.001
TW	7,088.34 (4,073.04, 11,140.37)	4,072.28 (2,371.38, 7,418.92)	1,873.11 (1,075.33, 3,854.96)	0.001
CEI	0.72 (0.65, 0.84)	0.57 (0.53, 0.62)	0.64 (0.60, 0.72)	<0.001
TEI	1.16 (1.10, 1.44)	0.99 (0.94, 1.06)	1.19 (1.00, 1.90)	<0.001
MMLRG	0.18 (0.00, 0.34)	0.04 (0.00, 0.25)	3.39 (2.11, 6.61)	<0.001

a Based on the independent-sample Kruskal-Wallis test.

CMI, case-mix index; ND, number of DRGs; TW, total weight; CEI, cost efficiency index; TEI, time efficiency index; MMLRG, mortality of middle and low-risk groups cases.

## 4 Discussion

### 4.1 Rationales and indicators for medical service performance evaluation

Since 2019, China has implemented a national initiative to strengthen performance evaluations of tertiary public hospitals, establishing a 55-indicator national evaluation framework as a reference for health authorities at all levels (35). In 2019, the Sichuan Provincial People’s Government launched a targeted evaluation of tertiary hospital performance (36), followed by the Sichuan Health Commission’s 2020 initiative for secondary public hospitals (37). These regional frameworks primarily relied on isolated health indicators, similar to historical approaches. In contrast, numerous studies have adopted DRG_S_ indicators to develop comprehensive evaluation models for assessing medical service performance across hospitals or regions (14, 18–21, 23, 25, 26, 28, 29, 38). Adoption of DRGs indicators addresses the limitations associated with single-index methods, the challenges in horizontal comparisons (10), and thus enhances evaluation efficiency (39).

### 4.2 Rationality of the DRGs-based evaluation model integrating EFA and HCA

Our study developed a novel evaluation model incorporating EFA and HCA using DRG_S_ indicators to evaluate 306 hospitals in Sichuan. The model’s scientific validity stems from two key strengths: data authenticity and methodological rigor. Hu et al. identified common issues in Chinese medical quality evaluations, such as unreliable data sources and low accuracy (40), which our study mitigates through DRGs indicators derived from the FPMR database (23). This ensures original data authenticity, consistency, and standardization, and thereby guaranteeing the credibility of the evaluation results.

Another notable issue concerning DRG_S_ indicators is multicollinearity, defined by Mamouei et al. as inter-variable correlations that distort statistical inference (41). Compared to traditional evaluation indicators, DRG_S_ indicators are interrelated and mutually constrained (10, 39). For instance, increasing CMI (by treating more severe cases) often elevates LOS and medical costs, thereby influencing CEI and TEI. To address this, our study adopted EFA to condense six original indicators into three significant CFs, reducing indicator interactions and enhancing evaluation reliability (10). This approach aligns with PCA methodologies recommended for multicollinearity mitigation (41).

### 4.3 Disparities in overall performances of the study hospitals

Significant disparities in the medical service performance of 306 study hospitals in Sichuan Province were observed in 2024. TGHs in the Chengdu region predominantly outperformed SGHs in northeastern Sichuan, findings contradict with the conclusion that minority-inhibited regions in Sichuan generally exhibited the worst performance (42–44), but partially consistent with previous studies (42, 45). These discrepancies may be attributed to the combined influences of hospital location and hospital level.

Geographically, Chengdu’s advantageous location, high economic development, strong government support (42), and advanced medical resources facilitate the attraction of skilled healthcare professionals (42, 45) and continuous enhancement of medical techniques. This enables the region to handle a larger patient volume, especially those with severe illnesses. Despite the high pressures of serving massive patient loads, hospitals here typically demonstrate higher management skills to maintain medical quality and retain their leading position in performance evaluations. However, as to northeastern Sichuan, their worst performance may be partially attributed to the underdeveloped economy, insufficient allocation of high-quality medical institutions (especially tertiary hospitals), and imbalanced healthcare talent structure. Relevant data show its GDP growth rate has been significantly lower than the provincial average, which may strain fiscal support for healthcare and hinder development (46). In 2022, tertiary hospitals accounted for only 11.4% in the region-far below the proportion in Chengdu, indicating a severe shortage of premium healthcare resources. Additionally, the region suffers a dearth of experienced practitioners, with only 9.2% of staff holding senior qualifications, far below the 38.7% in Chengdu (47, 48).

From the perspective of hospital level, China’s hospital system is categorized into three tiers (49). TGHs function as regional medical centers, integrating comprehensive capabilities in clinical care, education, and research to provide high-level specialized services across regions, cities, provinces, and even nationwide (50). Equipped with abundant medical resources and top-tier talent, they inherently achieve higher technical standards. Previous studies have also shown that tertiary hospitals dominate healthcare service provision in China, regardless of the severity of illness (51–53). Their large patient bases, advanced equipment, and skilled personnel confer a decisive advantage in performance evaluations. In contrast, SGHs primarily serve local communities while undertaking limited teaching and research roles (49). Compared to tertiary institutions, they lag in bed capacity, clinical department configuration, medical equipment, technical proficiency, talent reserves, and research capabilities (54, 55). These structural gaps place secondary hospitals at a competitive disadvantage, typically resulting in lower rankings in performance evaluations.

### 4.4 Disparities in performances among different clusters

#### 4.4.1 Performance variations in TGHs

Our study observed that the majority of tertiary general hospitals were clustered into the “Middle” cluster, with over 10% classified into the “Inferior” cluster. In 2021, the Health Commission of Sichuan Province issued the “Implementation Rules for the Evaluation Criteria of Tertiary Hospitals in Sichuan Province (2021 edition),” instructing tertiary hospitals in the region to enhance daily management and consistently improve medical quality (10). Despite uniform construction standards for THGs in Sichuan, significant disparities in medical service performance were revealed. Hospitals in the “Excellent” cluster significantly outperformed those in the “Middle” and “Inferior” clusters in medical ability and time efficiency (10, 42). However, they demonstrated poorer performance in cost efficiency (10, 42) and medical security, which contrasts with previous studies (10).

Hospitals in the “Excellent” cluster typically serve as top-tier regional institutions, benefiting from stronger government support, advanced medical equipment, and highly skilled healthcare teams. This enables them to treat a larger volume of patients, particularly those with severe illnesses, thereby expanding their disease coverage. As a result, they exhibit higher medical abilities, as indicated by higher CMI, ND, and TW values compared to other hospitals. Previous studies have found that regions generally exhibit lower time efficiency regardless of their medical service levels (42). However, our study identified that “Excellent” cluster hospitals performed best in time efficiency. This finding contrasts with previous research and may be attributed to their superior hospital management skills and professional teams. Health authorities have set stringent and uniform requirements on inpatient LOS in tertiary hospitals (14). Our findings suggest that “Excellent” cluster hospitals, with their specialized management teams and advanced management skills, can achieve better performance in time efficiency performance.

Consistent with previous studies (10, 42, 45, 56), our study found that “Excellent” cluster hospitals exhibited higher medical costs and mortality of middle and low-risk patients, likely linked to their status as regional top-tier institutions. These hospitals are responsible for admitting and treating regional severe, complicated, and acute cases (57), which consume more medical resources (e.g., advanced equipment and experienced staff), require longer hospital stays, and have higher treatment failure rates due to the severity of conditions. Collectively, these factors may contribute to the elevated medical costs and mortality values observed in “Excellent” cluster hospitals.

#### 4.4.2 Performance variations in SGHs

Our study revealed that, similar to TGHs, the majority of SGHs were clustered into the “Middle” cluster, with fewer than 5% clustered into the “Inferior” cluster. Unlike TGHs, SGHs in the “Excellent” cluster only outperformed in ND and TW. In contrast, those in the “Inferior” exhibited the best performance in CMI but the worst in MMLRG. Neither the “Excellent” nor the “Inferior” cluster hospitals performed best in CEI and TEI. These findings suggest that “Excellent” cluster hospitals outperformed in disease coverage and total inpatient output, whereas “Inferior” cluster hospitals performed relatively better in admitting and treating severely ill patients but struggled to maintain medical service quality, as evidenced by the disproportionately high MMLRG.

In 2019, the Health Commission of Sichuan Province issued the “Guidelines on Further Improving the Graded Diagnosis and Treatment System,” which explicitly stipulated the establishment of a scientific and efficient two-way referral mechanism between lower-level hospitals (secondary or grassroots) and tertiary hospitals within the region to meet the local medical service demands (58). Nevertheless, there are no mandatory regulations regarding patient referrals between SGHs. Patients may choose “Inferior” cluster hospitals based on geographic convenience rather than the hospital’s medical abilities for treating their conditions. As a result, these patients may be randomly admitted and treated by SGHs. The higher CMI values suggest that “Inferior” cluster hospitals may receive a great number of severe patients due to their convenient location. However, their medical abilities may fall short of the required standards for treating these patients, as indicated by the alarmingly high MMLRG values. Therefore, for SGHs in the “Inferior” cluster, enhancing medical abilities and maintaining medical quality should be prioritized as urgent tasks to ensure inpatient safety.

### 4.5 Limitations of our study

Several limitations of our study should be acknowledged. Firstly, although the data used in this study originated from the FPMR, potential errors may have occurred during the upload process to the SHDADSCP for generating DRG_S_ indicators. These potential errors could influence the results of the medical service performance evaluation. Secondly, the algorithms for calculating DRG_S_ indicators vary across different regions. For instance, BJ-DRG for Beijing and MS-DRG for Sichuan. Even with the same original data, the results of DRG_S_ indicators may differ due to these regional variations. Therefore, caution should be exercised when applying our findings to other regions. Thirdly, this study did not consider the weights of DRG_S_ indicators in constructing the performance evaluation model. Previous studies have demonstrated that different weights assigned to evaluation indicators can potentially affect the evaluation results (3). Therefore, future studies are anticipated to explore these underlying mechanisms.

## 5 Conclusion

DRG_S_ serve as a widely adopted risk adjustment tool for evaluating the medical service performance both within and among hospitals. By implementing EFA, our study mitigated the multicollinearity inherent in DRG_S_ indicators, yielding more reliable and accurate evaluation results. Significant disparities in medical service performance were observed across different regions and hospital levels in Sichuan Province, with Chengdu region demonstrating optimal performance. For TGHs, hospitals in the “Inferior” cluster are recommended to enhance their medical ability and efficiency compared to those in the “Excellent” cluster. Conversely, hospitals in the “Excellent” cluster should focus on controlling medical costs compared to those in the “Inferior” cluster. For SGHs, hospitals in the “Inferior” cluster should concentrate on improving medical security and ensuring patient safety compared to those in the “Middle” and “Excellent” clusters.

## Data Availability

The raw data supporting the conclusions of this article will be made available by the authors without undue reservation.
